# Improving total body irradiation with a dedicated couch and 3D-printed patient-specific lung blocks: A feasibility study

**DOI:** 10.3389/fonc.2022.1046168

**Published:** 2023-01-19

**Authors:** Silvia Strolin, Giulia Paolani, Miriam Santoro, Laura Cercenelli, Barbara Bortolani, Ilario Ammendolia, Silvia Cammelli, Gianfranco Cicoria, Phyo Wai Win, Alessio G. Morganti, Emanuela Marcelli, Lidia Strigari

**Affiliations:** ^1^ Department of Medical Physics, IRCCS Azienda Ospedaliero-Universitaria di Bologna, Bologna, Italy; ^2^ eDIMES Lab-Laboratory of Bioengineering, Department of Experimental Diagnostic and Specialty Medicine, (DIMES), Alma Mater Studiorum University of Bologna, Bologna, Italy; ^3^ Radiation Oncology, IRCCS Azienda Ospedaliero-Universitaria di Bologna, Bologna, Italy; ^4^ Department of Experimental, Diagnostic and Specialty Medicine-DIMES, Alma Mater Studiorum University of Bologna, Bologna, Italy

**Keywords:** total body irradiation, hematopoietic stem cell transplants, 3D-printing, lung shielding, treatment planning system optimization

## Abstract

**Introduction:**

Total body irradiation (TBI) is an important component of the conditioning regimen in patients undergoing hematopoietic stem cell transplants. TBI is used in very few patients and therefore it is generally delivered with standard linear accelerators (LINACs) and not with dedicated devices. Severe pulmonary toxicity is the most common adverse effect after TBI, and patient-specific lead blocks are used to reduce mean lung dose. In this context, online treatment setup is crucial to achieve precise positioning of the lung blocks. Therefore, in this study we aim to report our experience at generating 3D-printed patient-specific lung blocks and coupling a dedicated couch (with an integrated onboard image device) with a modern LINAC for TBI treatment.

**Material and methods:**

TBI was planned and delivered (2Gy/fraction given twice a day, over 3 days) to 15 patients. Online images, to be compared with planned digitally reconstructed radiographies, were acquired with the couch-dedicated Electronic Portal Imaging Device (EPID) panel and imported in the iView software using a homemade Graphical User Interface (GUI). *In vivo* dosimetry, using Metal-Oxide Field-Effect Transistors (MOSFETs), was used to assess the setup reproducibility in both supine and prone positions.

**Results:**

3D printing of lung blocks was feasible for all planned patients using a stereolithography 3D printer with a build volume of 14.5×14.5×17.5 cm^3^. The number of required pre-TBI EPID-images generally decreases after the first fraction. In patient-specific quality assurance, the difference between measured and calculated dose was generally<2%. The MOSFET measurements reproducibility along each treatment and patient was 2.7%, in average.

**Conclusion:**

The TBI technique was successfully implemented, demonstrating that our approach is feasible, flexible, and cost-effective. The use of 3D-printed patient-specific lung blocks have the potential to personalize TBI treatment and to refine the shape of the blocks before delivery, making them extremely versatile.

## Introduction

Total Body Irradiation (TBI) is an important component of the conditioning regimen in patients undergoing hematopoietic stem cell transplants. In particular, the combination of radiotherapy and chemotherapy may achieve greater tumor cytotoxicity and can improve the spatial distribution of therapeutic effects (1–4). Indeed, TBI is able to: i) destroy cancer cells in areas (such as the nervous system, bones, skin, or testes) not easily reachable by chemotherapy; ii) inhibit the response of the immune system before the allogeneic stem cell transplantation (bone marrow or stem cells from a donor), and thus iii) allow the transplanted bone marrow to grow (engraft) (5).

TBI aims to deliver a uniform dose of ionizing radiation throughout the body. Considering the non-standard size of the target, different technological solutions have been developed in recent years to optimize TBI schedules, patient positioning, beam dimensions, and low dose rate through increased source‐to‐surface distance (SSD) (6–9). Traditionally, intensity modulated radiation therapy (IMRT) with large treatment fields and gantry angled at 90° or 270° are used to simultaneously irradiate the entire target by lateral or anterior-posterior (AP) beams with patients set in a fetal position near the bunker wall or on the bunker floor using dedicated couches to accommodate the entire patient within the radiation field. Lateral solutions provide higher dose homogeneity but reduced patient comfort and limited possibility of Organs at Risk (OARs) shielding, mainly constituted by lungs (10, 11). Indeed, lung toxicity is the most common adverse effect after TBI with a rate covering a range of 10.3–45%, depending on the patient cohort and treatment technique (12–20). Therefore custom-shaped lead blocks are designed and realized for each patient and treatment position to partially shield the lungs but not the target volume. Unfortunately, lung blocks are expensive and not reusable. However, in recent years the use of 3D printers in healthcare has been tested with growing interest given their ability to produce complex and customizable forms (21), even reproducing the complexity of human anatomy (22–28). In particular, 3D printing has been recently applied in radiotherapy, mainly to produce patient-specific immobilization devices (28).

Thanks to the possibility of modeling also extended SSD with treatment planning system (TPS), Kirby et al. (29, 30) developed the inverse-planned single modulated sweeping arc therapy TBI technique (MATBI) to overcome AP/PA beams limitations. MATBI is based on several static 40×40cm^2^ radiation fields with different number of monitor units (MUs), distributed over the patient’s length and combined to produce an arc to mimic a Volumetric Modulated Arc Therapy (VMAT). In addition, Jahnke et al. (31) used single modulated sweeping arc therapy to deliver the planned dose with a single gantry rotation and the patient lying on a dedicated bed close to the bunker floor, in supine and prone position. Moreover, Pierce et al. (32) provided a VMAT solution using the multi-leaf collimator (MLC) modulation to reduce the dose to the patient’s periphery and lungs. Beam weights were defined based on Jahnke et al.’s work (31) and adjusted to improve dose homogeneity using the dose calculated on patient CT scans using the AAA algorithm at extended SSD. Indeed, a major concern in planning TBI is the dose homogeneity throughout the body outside the lungs which should be within 10% of the prescribed dose (33).

The use of Gafchromic EBT3 films (34–36), EDP-30 diodes (37), and other semiconductors (38) are considered effective to perform accurate and timely *in vivo* dosimetry. Compared with other dosimeters, Metal-Oxide Field-Effect Transistor (MOSFET) has the advantage of a direct dose readout and a good linearity response without significant variation with temperature and/or accumulated dose, already used for TBI (39).

Setup positioning represents a central issue in TBI treatments due to the dose delivered to the whole body and to the importance of precise positioning of lung blocks. In particular, the availability of online images is crucial to implement an effective and safe procedure by monitoring several potentially critical aspects. In addition, patient setup is a time-consuming procedure potentially limited by patient compliance or sedation duration, especially in case of pediatric patients.

To renew and improve the TBI technique carried out in our center, we launched a fund-raising project with the support of the Fondazione Sant’Orsola, which financed the acquisition of a dedicated TBI treatment couch. Moreover, we introduced in our workflow, the manufacturing of patient-specific 3D-printed lung blocks to improve treatment accuracy and to reduce the cost of *ad-hoc* produced lead shields.

Therefore, this manuscript aims to report the main steps of a workflow aimed at generating 3D-printed patient-specific lung blocks and integrating a dedicated couch with a modern LINAC for TBI treatment.

## Materials and methods

### Dedicated couch

**Figure 1:**
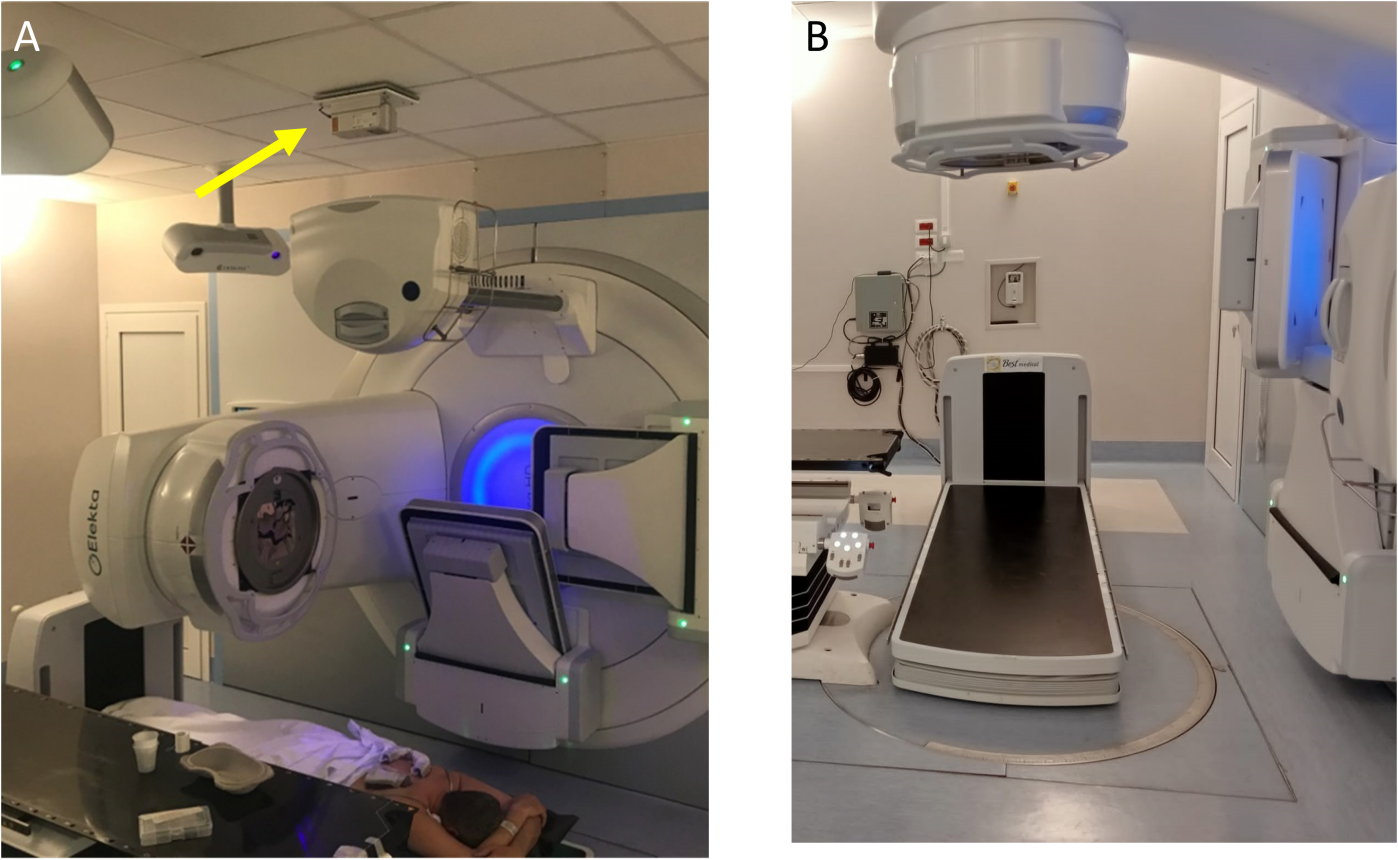
**(A)** Pt#1 in prone position on the dedicated couch coupled with a VERSA HD Linac (Elekta, Stockholm, Sweden). A dedicated additional laser, visible on the bunker’s ceiling and indicated with yellow arrow in panel **(A)**, enables the patient alignment when placed on the TBI couch. With the gantry rotated 270°, the cone beam-CT tube allows the delivery of a kV X-rays beam for the setup imaging. At the end of this procedure, the cone beam-CT tube is retracted, and the gantry is rotated 0° for the TBI delivery in the prone position. **(B)** Positioning of dedicated TBI couch for patient treatment with Elekta couch retracted.

In this work, the dedicated couch of GammaBeam 500 Total Body Irradiator described in (40) has been used. The system has a motorized vertical motion to allow easy patient access before lowering the couch to the treatment position. Moreover, it has a 41x41cm a-Si flat-panel imager with a motorized longitudinal motion for patient and lungs blocks setup checks. Both setup images acquisition and treatment delivery can be performed when the couch is at its lowest position, with the tabletop 18 cm above the floor. A blocking tray can be attached to the couch to hold custom lung blocks close to the patient skin at three standard heights (19, 27, and 34.7cm) above the treatment couch using an accessory trail. To enable other heights (22.2, 24, 28, 29.7, 31.5, 35.5, 37.2, and 39cm), additional connectors of the blocking tray to the couch were designed and 3D-printed using a stereolithography 3D printer (Form3B, Formlabs). A photograph of the unit coupled with a VersaHD LINAC released by *Elekta* (Elekta, Stockholm, Sweden) is shown in **Figures 1A, B**.

With this configuration, the typical distance from the source to the surface of the treatment couch is 206.5 cm. During the commissioning process, as described in (40), we evaluated the operational accuracy of all mechanical systems of the couch and of the imaging system and we optimized the acquisition parameters of the kV tube, in order to couple the couch with a modern conventional linear accelerator.

### Patients

Fifteen patients have been successfully planned and treated. Details on the plan implementation using treatment planning system are reported in Supplementary Material (subsection “Treatment Planning System implementation (LINAC and dedicated planning CT)”). There were 7 males (46.7%) and 8 females (53.3%) with a median [range] age of 36 years (7–55). The main patients’ characteristics are reported in **Table 1**.

**Table 1:** Patient characteristics.

*Pt #*	*Sex*	*Age (yrs)*	*Weight (kg)*	*Height (cm)*	*Lung volume (cc)*	*Body volumes (cc)*	*Heart volumes (cc)*
*1*	M	43	88	171	3462	91487	1072
*2*	F	55	61	157	1917	61498	573
*3*	F	22	80	161	1914	84295	656
*4*	F	55	64	167	2397	69726	654
*5*	M	19	89	175	2329	94228	770
*6*	F	38	52	169	2730	52584	382
*7*	M	53	84	176	3032	84949	675
*8*	M	17	75	189	3358	63903	675
*9*	F	7	24	135	1018	27189	263
*10*	F	29	90	172	1874	77963	650
*11*	M	36	93	178	2788	91213	778
*12*	F	44	62	165	3023	58486	464
*13*	F	46	70	160	2993	69140	327
*14*	M	28	56	167	2623	52050	358
*15*	M	15	44	176	1583	44290	317

M, male; F, female.

The immobilization system was realized using two *Vac*-*Lok*™ *Cushions*, creating a rigid and secure support around the patient when a vacuum is drawn through a self-sealing quick-release valve. The patient was positioned with or without the arms above the head to improve comfort and to accommodate him/her on the couch, with a maximal length of about 185 cm. The first Cushion was fitted to the patient in supine position while the second Cushion (with a cavity to accommodate the patient’s face) was placed above the patient (in supine position), fitted, and modeled to realize a flattened upper surface, and was used for CT acquisition and treatment in prone position.

These Cushions retain their shape and guarantee stability and reproducibility of the patient’s position for up to six weeks once the air is vacuumed out. The treatment area is artifact-free with minimal beam attenuation, and Cushions are reusable after sanitization.

### Contouring and design of patient-specific 3D-printed lung blocks

**Figure 2:**
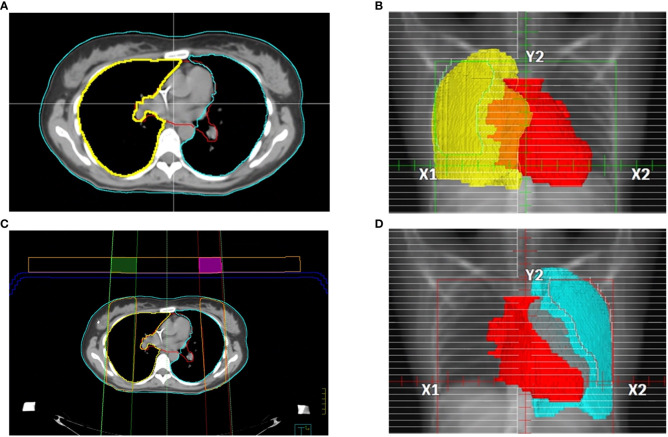
Main steps of lung blocks design: **(A)** example of axial CT images with several representative contours, in details described in the manuscript. Pinnacle BEV window for **(B)** right and **(D)** left lung block design. **(C)** block tray (in blue) and 3 cm thickness parallelepiped ROI with the right and left block beams, 60% isodose line (in orange). The intersection of the of contours generated by the 60% isodose line and the 3 cm thickness parallelepiped ROI produced the right (green) and left (purple) lung blocks to be 3D-printed.

The CT acquisition was performed using a PET/CT scanner Discovery MI (GE-Healthcare) with 120 kV with 48 axials plus 721 helical images with a minimum slice thickness of 2.5mm. Images were reconstructed with a thickness of 5 mm to decrease the total time for TPS calculation. The contouring included: lungs, liver, clavicles, heart, and bilateral iliac bones (**Figure 2**). In both supine and prone block trials, two fixed beams right and left with gantry angle of 0° and the same isocenter of TBI plan were created and visualized in the Beam’s Eye View (BEV) window using Pinnacle TPS. Radiation oncologist, in each fixed block beam, separately contoured the area of right and left lung to be shielded, avoiding liver dome, clavicles, and heart, representing, among others, treatment target volumes (**Figures 2B, D**). A margin of 5 mm from the projection of these target volumes was guarantee during the lung block drawing. In each fixed block beam, the MLC leaves were used to expose each block (i.e., the right and left lung to be shielded).

To convert the exposed area into 3D lung blocks, we adopted two strategies. In case of adopting the blocking tray for the treatment, both the blocking tray and a parallelepiped ROI with a height of 3 cm were loaded as structures in Pinnacle and moved in the antero-posterior direction at the more appropriate height from the couch’s surface by considering the patient thickness and the available heights of the blocking tray, to minimize the air gap between the tray and the patient surface. In case of 3D lung blocks directly placed on patient skin, an expansion of the body contour of 3 cm on the anterior direction was performed. After that, a calculation grid (grid dimensions: 4x4x4 mm^3^) around the parallelepiped ROI was inserted, and the 60% isodose was converted into the right or left lung block. The 60% isodose was chosen because it provided the optimal block shape for sparing the contoured lung area. This isodose was converted in ROI included in the parallelepiped area or in the expanded body using Pinnacle Boolean functions for ROIs (**Figure 2C**)

**Figure 3:**
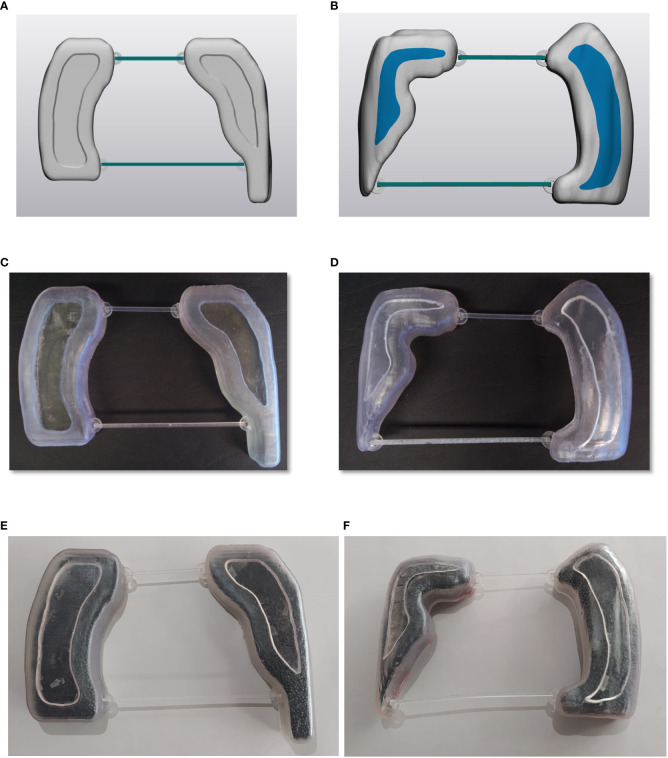
Panels **(A, B)** illustrate an example of digital lung blocks for supine and prone set-up, respectively, panels **(C, D)** show the hollow containers of 3D-printed lung blocks while panels **(E, F)** report the containers filled with lead spheres.

The planned lung blocks were imported as DICOM format files into 3D Slicer segmentation software and then converted into Standard Tessellation Language (STL) for further CAD (Computer Assisted Design) operations. The lung blocks were designed as hollow containers of 2 mm wall thickness with a cover, using 3-matic software (Materialise NV, Leuven, Belgium) as shown in **Figures 3A, B**. Two removable spacer bars were also designed to maintain the correct relative distance between blocks. All the parts were exported in STL format to be printed with a stereolithography (SLA) 3D printer (Form 3B, Formlabs, Somerville, MA, USA) using a photosensitive clear rigid resin (density: 1.03 g/cm^3^). A representative example of printed hollow containers is reported in **Figures 3C, D**.

**Figure 4:**
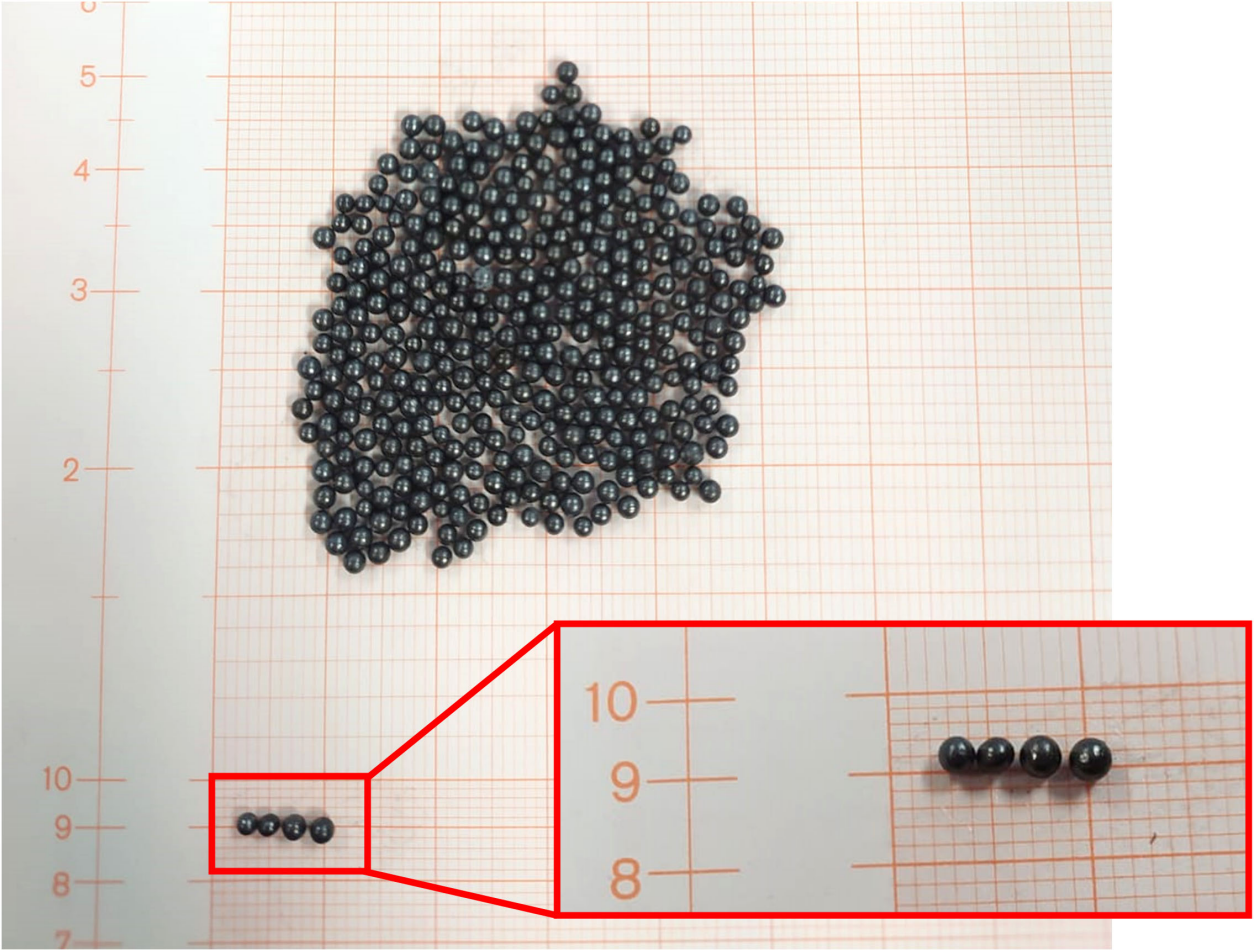
Lead spheres used to fill the 3D-printed allow lung blocks. Graph paper was used to point out the spheres dimension.

Finally, the lung blocks were filled with lead spheres (**Figures 3E, F**), having a density of 6.81 ± 0.14 g/cm^3^ and a median [range] diameter of 1.5 mm [1.2-2.0]. The actual density depends on the number and size range of lead sphere, which were taken into consideration both in the final dose calculation and *in vivo* measurements. A representative image of the used lead sphere is reported in **Figure 4**.

**Figure 5:**
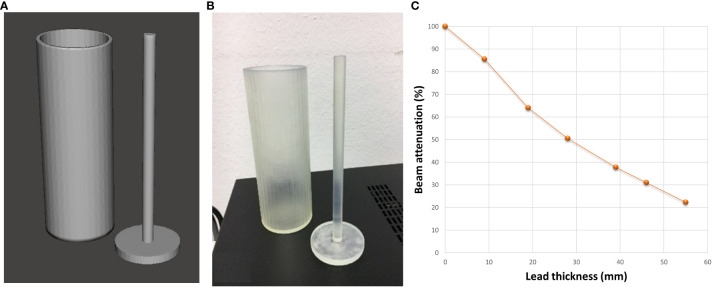
Digital **(A)** and 3D-printed **(B)** models of the *ad-hoc* cylinder used to determine the filling level of the lung blocks with lead spheres. **(C)** Photon diode-measured % beam attenuation against the thickness of cylinder filled with lead spheres.

An *ad-hoc* cylinder was designed, and 3D-printed to determine the filling level of lead spheres according to the desired attenuation for the patient-specific blocks (**Figure 5**). The electronic density determined using this cylinder filled with lead spheres was included in the TPS software to define the height of the parallelepiped ROI for generating the lung blocks.

### Development of treatment planning

TBI was delivered with 2Gy/fraction given twice a day, over 3 consecutive days (total dose: 12Gy). The treatment was planned using Pinnacle^3^ TPS version 16.2 (*Philips* Medical Systems, Fitchburg, WI, USA) and was based on 48 beams with a gantry angle from 315° to 72.5°, a field size of (40x25-30) cm^2^ (varying following the largest lateral dimension of the patient), and beam angle spacing of 2.5°. For all the patient, the isocenter was placed on the patient’s skin at 2 cm from the sternum in the caudal direction. This configuration was applied to the CT images of patients in prone and supine position.

Beam weighting factors were determined using inverse planning beam weighting optimization using all the beams (of the anterior and posterior plan) on the supine CT images with the aim of delivering a uniform dose to the planning target volume (PTV) while sparing the lungs (29).

During the beam weighing optimization phase, the overridden densities of both the block tray (electronic density: 0.8 g/cm^3^) and 3D-printed lung blocks (electronic density: 8.9 g/cm^3^) were considered to achieve a homogenous dose distribution in non-spared tissues. The final fine-tuning of the plan in the TPS allows better coverage of volumes with higher risk of treatment failure. The beam weights calculated for supine plan were applied to prone CT images. The prone and supine 3D dose distributions were summed using MIM Vista software version7.1.4 (MIM Software Inc., Beachwood, OH, USA), adopting an *ad-hoc* workflow based on the AAPM TG 132 (41).

If necessary, beams with proper dimensions were added to improve dose homogeneity in case of under dosage of the abdomen or lymph nodes surrounding the clavicles. In case of patients with arms along the body, the fields dimensions in latero-lateral direction were reduced to decrease the arm overdosage.

Thus, a final tuning was performed separately for each plan up to guarantee a homogenous dose distribution. The (body–lungs)-5mm volume of interest was used to refine the optimized treatment plans and their sum, thus, to assess the target dose homogeneity index (42) as follows:


$$Homogeneity\;Index=\frac{D(2\%)\;-\;D(98\%)}{D(prescription)}$$

Thereafter, the DVHs of the accumulated plans, in both supine and prone position, were generated, discussed with the radiation oncologist, and finally approved. The approved plans were sent to the MOSAIQ record and verify (R&V) system. Thus, all beams were linked to generate a single modulated sweeping arc for both supine and prone approach based on automated beam sequence (31).

### Pre-treatment quality assurance (QA)

**Figure 6:**
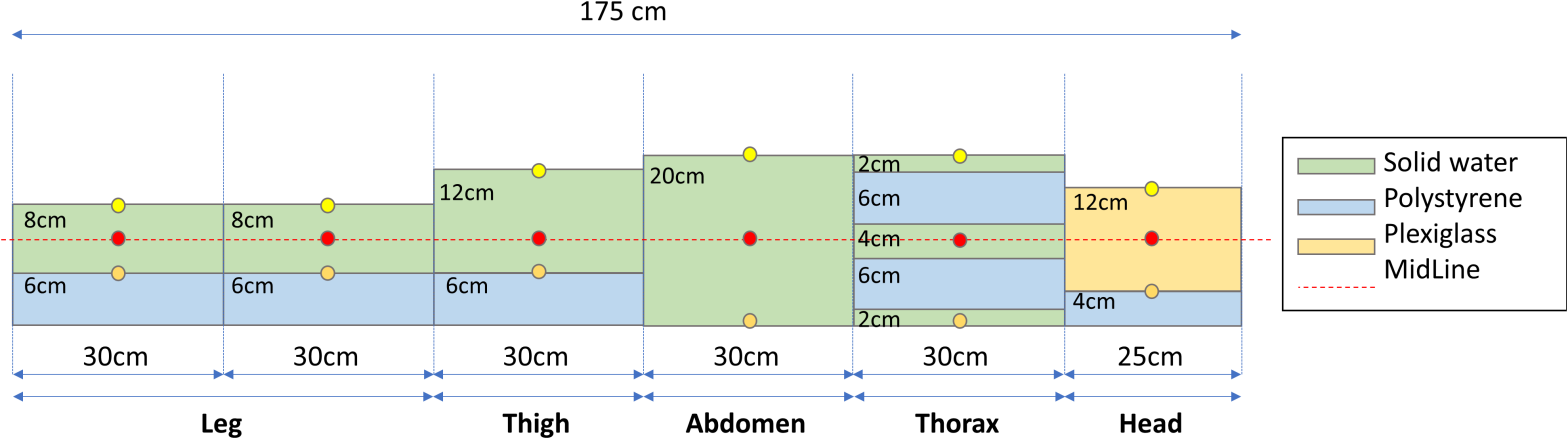
An *ad-hoc* phantom was created using different water-equivalent plastic slab phantoms to simulate an example patient. Circles indicate measurement points using the farmer at entrance (yellow), midline (red), and exit (orange) position.

Before treatment delivery, a patient QA plan was performed using an *ad-hoc* homemade phantom of water-equivalent plastic, polystyrene, and plexiglass slabs. The phantom was designed to reproduce the body densities as reported in **Figure 6**. The QA plan was prepared with the phantom without density override to both the accessory support tray and attenuators. Farmer measured and calculated data were determined in several points of the dedicated phantom as indicated in **Figure 6** (at 1.3cm from the phantom surface or at the center of slabs through the whole phantom).

### Patient’s setup imaging

The TBI couch-dedicated Electronic Portal Imaging Device (EPID) panel was used to verify the lung block positioning prior to treatment on the patient skin or block tray and compared with the Pinnacle generated Digitally reconstructed radiographs (DRRs) (see **Supplementary Material Figure S1**). The kV tube of the X‐ray volume imager (XVI) system (Elekta, Stockholm, Sweden) was used to generate the set-up imaging with the EPID panel included in the TBI couch, while placing the XVI panel in the rest position (see **Figure 1**). The reference values of XVI panel position were properly changed using the *ad-hoc* developed file saved as sri.ini. In the original configuration, the couch dedicated EPID panel was coupled with the Co-60 beam, so the combination of mA and seconds was optimized to reach the appropriate quality for the setup imaging by using a thorax phantom to mimic the site in which the lung blocks are placed. Thus, a TBI setup has been added to the imaging kV protocol of XVI software. The number of acquired images per session and patients were registered and analyzed according to the use of the block tray. The images in.his format cannot be imported in the iViewGT software without conversion in.jpeg format. Details on the image conversion procedure is reported in Supplementary Material paragraph “Patient’s setup imaging and conversion” Thus, the converted file can be imported into the iViewGT imaging software to be compared with the plan-generated DRR, for verifying the actual lung blocks displacement. The position of each block is visually checked, eventually manually adjusted by the radiation oncologist and further verified using imaging as above described.

### *In vivo* dosimetry

*In vivo* measurements were performed using five MOSFETs and EBT3 Gafchromic films to evaluate the dose at the entrance and exit under the blocks and at the entrance of patients at 15cm from the isocenter, in caudal direction. For MOSFET placed at patient entrance a printed 1-cm cup of build-up was used, while the Gafchromic was partially placed under the MOSFET and its build-up cup. The distance of 15 cm from isocenter was considered as a surrogate of abdominal/pelvis entrance dose, slightly influenced by lung block position. The dose obtained using MOSFET, and Gafchromic films were also analyzed and compared. The reproducibility of measurements was investigated considering the standard deviation of values collected in the six fractions in supine and prone positions for each patient. The MOSFET measurements’ reproducibility was considered as a surrogate of the accuracy of the delivered dose. The median [range] of a standard deviation per session was calculated.

### Acute toxicities

TBI-induced acute toxicity was daily monitored and scored using the National Cancer Institute Common Terminology Criteria for Adverse Events (CTCAE) v.5.0. The follow-up of treated patients is still ongoing to evaluate treatment outcomes and late toxicity.

## Results

### Pre-treatment QA and measurements

All plans were successfully implemented in the TPS and verified using the Farmer chamber at the reference points described in the Materials and Methods section and reported in **Figure 6**. The agreement between considered points was<2% with the unique exception of one point measurement close to the control bar including the motor for vertical movement. Indeed, the metallic components included in the head of the couch placed in correspondence of patient feet (see **Figure 1**) may increase the scattered radiation increasing the experimental measurements of absorbed dose of 1-2% when compared to TPS-based one. In addition, the shielding capability of 3D-printed lung blocks was checked using Gafchromic films due to the typical dimension of lung blocks (<9cm in latero-lateral direction), for pediatric patients. The ratio between standard deviation and mean absorbed doses collected using Gafchromic films placed along the latero-lateral direction and partially under the 3D-printed lung blocks revealed a homogeneous filling of the blocks and confirmed their attenuation during the patient pre-treatment QA and/or during treatment.

### Acute toxicity

2/15 patients developed Grade 1 xerostomia, 6/15 patients developed Grade 1 nausea and 4/15 patients developed Grade 1 asthenia. All these acute toxicities were reported on the last treatment day. All patients had Grade 2-3 leucopenia, mainly due to the previous chemotherapy conditioning regimen. No patient developed diarrhea and/or vomiting. No radiation dermatitis higher than Grade 1 or acute gastrointestinal toxicities were observed.

### Use of 3D-printed lung blocks on patients

**Figure 7:**
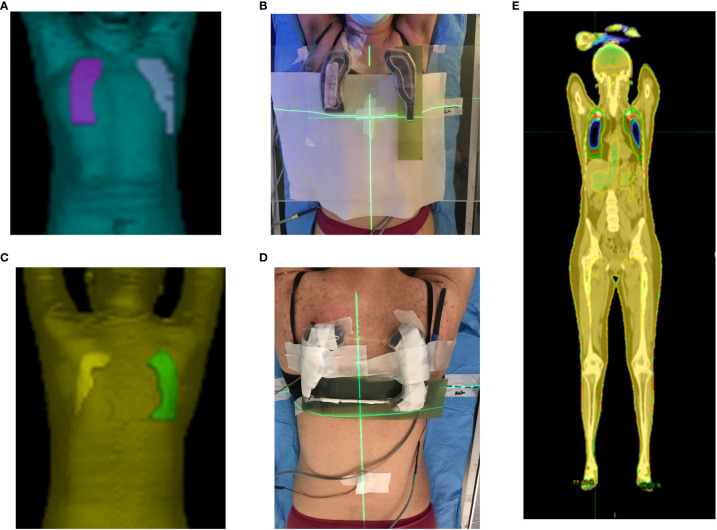
Planned **(A, C)** and printed **(B, D)** lung blocks for the supine **(A, B)** and prone **(C, D)** treatment position. The supine lung blocks were placed over the dedicated block tray while those for prone position setup were placed on patient skin. MOSFETs and Gafchromic films are also shown in panel **(B, D, E)**. Accumulated dose distribution on the supine images.

Before using the lung blocks on patients, the percentage depth dose (PDD) below the cylinder filled with lead spheres was evaluated against the shielding thickness in mm (**Figure 5C**). In our cohort of patients, a thickness of lead spheres of 3 cm was considered appropriate for the supine (**Figures 3A**, **7A** on patient skin) and prone (**Figures 3B**, **7C** on patient skin) lung blocks placed on patient skin (**Figures 1**, **7D**) or over the dedicated block tray (**Figure 7B**), according to patients’ characteristics. As reported in **Figure 5C**, lung blocks with 3 cm of thickness filled with lead spheres allowed a beam attenuation >50%, making feasible the lung mean dose constraint ≤10Gy, adopted in all the patient with the exception of one to guarantee the target coverage.

In 2 patients the blocks were placed directly on the skin, in 11 patients the block tray was used for both the supine and prone position, and in 2 patients the block tray was used only in the prone position.

### TPS calculation and *in vivo* dosimetry

The mean doses obtained from TPS measurements to whole body, whole body minus lungs, heart, liver, and clavicles per fraction are shown in **Supplementary Material** (**Table S1** **)**.

The treatment plan objective was to achieve a mean dose of 12Gy to the PTV while reducing the mean dose to the lungs, to achieve a mean dose up to 10 Gy. In one patient, a mean lung dose of 10.7Gy was accepted to allow the delivery of the prescribed dose to the whole bilateral iliac bones.

Using the summed dose distribution calculated with MIM (example in **Figure 7E**), the median [range] dose homogeneity of (body–lungs)-5mm was 0.42 [0.24-0.74], while for lung dose it was 9.9 Gy [8.1-10.7]. The difference between measured and calculated dose using the pre-treatment patient-specific QA phantom was<2% for most of the points, except for points close to the head of the couch due to the scattered radiation (<3%).

The number of setup images collected before treatment ranged in average from 2.3 to 1.7 as shown in **Supplementary material Table S2**.

The average [range] of MOSFET measurements reproducibility along each treatment and patient was 2.7% (1%-9.4%). The variability of MOSFET measurements along the treatment was larger for MOSFETs placed at the exit of patients under the blocks and lower at the entrance of patient under the blocks or under the 1-cm cup of build-up. The Pearson correlation between MOSFET and Gafcromich films was 0.85 (p-value<0.001). Additional results on MOSFET and Gafchromic calibration and patient-specific *in vivo* measurements will be included in a separate manuscript, under preparation.

## Discussion

### TPS calculation

Thanks to the implementation of lasers on the PET/CT scanner, we imported the acquired images in Pinnacle without using the concatenation tool (29).

Many authors reported commercially available TPSs capable of determining the dose with an accuracy of 3% (29, 43, 44). Penumbral characteristics during the beam model in TPS played a vital role in accurate dose calculation and required the acquisition of measurement in the TBI setting. The PDD and profile agreed within 2% for most beam sizes up to 28x40cm^2^ with the exception of 40x40cm^2^, due to the maximal dimension of the water tank used for data collection (requiring an offset of the tank relative to the central axis) likely affected by the radiation scattered from the Elekta LINAC and couch. The calculated homogeneity index was higher than the mandatory value (0.5) in four patients due to underdosage of clavicles in proximity of shielded areas. Of note, the homogeneity index was calculated using (body-lungs)-5mm ROI to take into consideration the dose distribution gradient around the lungs and close to the body surface sampled using a calculation grid of 4x4x4 mm^3^, chosen as compromise between calculation time and required accuracy. The possible expected increased lung toxicity risk due to the target coverage was considered acceptable, considering the reduction of dose rate obtained with sweeping-arc (see the following subparagraph “4.7 Dose rate”). Finally, the disagreement of 1-2% between measured and calculated absorbed doses due to the metallic components included in the couch head might be reduced in the future by a more accurate modeling of the entire couch.

### Treatment delivery

The sweeping arc technique was feasible without using the spoiler because the use of contra-opposite and angulated beams (gantry angles higher than ±40°) increases the entrance dose to the patient skin, allowing a homogenous treatment, as reported in (31, 45).

The dosimetric requirements for TBI [AAPM Report 17 (33)] recommend a dose homogeneity within ±10%. The homogeneity of our approach was significantly improved (HI=0.43% in the summed plans) when compared to previous techniques, characterized by dose inhomogeneity up to 30%, in particular for overweighted patients treated with latero-lateral beams (46).

### Sweeping arc techniques

The TPS allows to import the sweeping arc technique and perform an automized beam weight optimization (29, 30) after the positioning of virtual lung blocks (i.e., the block structures with override density) in order to obtain a homogenous dose profile along the cranial-caudal- at the mid-plane of the patient.

In addition, the use of blocking tray highly increases the patient comfort, but is a crucial issue as the distance between blocks and patient’s surface increases the penumbra effect (47) at the clavicles and other crucial sites for disease relapse. Therefore, one or two additional fields were needed to improve target coverage with a minimal dose ≥95% of the prescribed dose. Of note, in the case of over or underweighted patients, additional connectors of the blocking tray to the dedicated couch were designed and 3D-printed to minimize the air gap between blocking tray and patient skin.

The possibility of using MIM for the ROI-based dose accumulation enables the fine-tuning of planning in the supine and prone positions. Overall, the possibility of using the TPS improved the dose homogeneity into the target, which was lower or similar to the one reported in other TPS-based TBI (i.e., generally<10%) (48).

### Setup

In our experience, regarding the setup and treatment delivery, the use of the dedicated couch with an on-board imaging device improves the setup verification phase by decreasing the session duration and improving patient comfort. In fact, the setup procedure was quick, typically taking less than 5 minutes for the prone and supine sessions. Obviously, the plan optimization relies on reference CT images, while involuntary patient movements (respiration, cough, isocenter shift, etc.) might impact the actual dose delivery. Therefore, compromise between treatment time and dose rate needs to be found. However, the collected images might be helpful in the future to model the hepatic dome movements and to better assess the uncertainties due to the respiratory movement.

### Patient-specific 3D-printed lung blocks

The novel approach of manufacturing patient-specific 3D-printed lung blocks demonstrated to be feasible and viable for its routine use in TBI. The use of 3D printing for blocks production in the TBI treatment has recently been described (49). However, in that case, 3D printing was mainly used to fabricate the photon block molds to be filled with MCP96 alloy: this approach is not always applicable, as the necessary technologies are generally not available, not affordable, and not allowed in hospital environment, due to safety issues. In our experience, lung blocks were designed, and 3D-printed directly as hollow containers, later filled with lead spheres.

In terms of costs, our solution is sustainable since the blocks can be produced using an affordable professional-grade 3D printer. Surely, also the material costs (mean: 40 euro/case) and the man-hours for CAD design (approximately 1 hour) must be considered. In addition, in our study, all blocks were printed as a single piece thus suggesting that their size is quite compatible with the printing bed and build volume of the printer. Moreover, the lead spheres poured in the patient-specific blocks can be re-used. Overall, the proposed approach allows for a cheaper (about 100 times) solution than conventional lead plates that have to be cut according to the patients’ lungs, and that are typically provided by specialized health care companies. Moreover, our workflow may offer the opportunity to carry out all planning phases within the hospital environment, clearly reducing the TBI treatment waiting time.

Another clear advantage of producing patient-specific 3D-printed blocks is their versatility. Indeed, in two of the treated patients, the *ad-hoc* settings allowed to change the portion of the blocks filled with the lead spheres using a printed septum. This septum was introduced after the first set-up images where a mismatch between DRR and verification imaging occurred, due to the different respiratory phase. From another point of view, the block thickness cannot be modified during the TBI procedure, but the cost and the printing time may allow the adaptation of blocks from one session to another or, alternatively, the printing of two sets with different thickness before treatment starting. These adjustments are much less complex and time consuming compared with modification of conventional lead blocks.

### Monitor units

Also, the number of MUs per session was lower compared to the one reported in experiences based on MLCs, despite achieving the prescribed dose in midplane due to the use of large fields (45). In our study, the treatment required about 1500 MU delivered in around 12 minutes for both prone and supine sessions, after a short learning curve (of about 4 treatments). In particular, the mean ± standard deviation of MUs for the supine TBI treatment were 1472 ± 200 MUs in our cohort of patients while they were 3456 ± 264 in (45), considered as a representative paper of MLC-modulated TBI treatment.

### Dose rate and toxicity

The dose rate plays a critical role in optimizing the TBI treatment time and to reduce treatment-related toxicity. In fact, interstitial pneumonitis is the major cause of mortality (up to 50% in myeloablative TBI) (29) but lung toxicity can be reduced by keeping the mean lung dose below 8-10Gy with a dose rate not exceeding 0.20Gy/minute (10, 50). Of note, the calculated dose rate in our study is intended at the entrance of the 3D-printed lung blocks. Using the proposed set-up, patients could remain in a fully stretched, comfortable position, and the beams are delivered with a dose rate of 400 MU/minute at the isocentre (100cm) but with a maximum effective dose rate of about 0.17 Gy/minute at the midline of the patient. This approach should reduce the expected lung toxicity (e.g., from about 11% to 2.3% using lung blocks (51) for a regimen of 12Gy in 6 daily fractions) in agreement with the guidelines from the International Lymphoma Radiation Oncology Group (10).

Nevertheless, this topic is still a matter of debate because lung-sparing may adversely affect the TBI efficacy. As under-dosage to sanctuary sites would increase the risk of relapse, no other organs except part of the lungs were shielded in our study. It is important to note that, in several centers, most shielded tissues (such as sternum and vertebrae) receive approximately 60-80% of the prescribed dose (51) and that chemotherapy administered during the conditioning regimen increases the therapeutic effect in these shielded areas. In our study, to avoid this unwanted dose reduction, one or more boost fields were added if necessary to avoid under-dosage on these sanctuary sites.

### *In vivo* dosimetry

The variability of MOSFET measurements along each treatment was larger in the first patients and at the exit of patient under the blocks, thus highlighting the need for a learning curve to accurately position the MOSFETs in points identifiable on calculated dose distribution (30). *In vivo* dosimetry permitted us to monitor treatment reproducibility and check correct lung block positioning, but the absolute values might be affected by the electron contamination at extended SSDs and larger gantry angles. This aspect is fully investigated in a separate paper under preparation.

### Total marrow irradiation and total marrow and lymphoid irradiation

Other treatment approaches, such as Total marrow irradiation and total marrow and lymphoid irradiation, are still under investigation as strategy to further reduce OAR toxicities with an improved disease control when compared to TBI and/or chemotherapy-alone conditional approaches (10). Also in this setting, our approach could be adapted taking into consideration the possibility of performing a pre-treatment image verification using the on-board EPID device and fully modulate the delivered dose by using the LINAC MLC. However, this potential application is out of the scope of this work.

### Possible treatment improvements

Other authors have chosen to decrease the dose rate for beams contributing to lung doses, to reduce radiation-induced lung toxicity, while they increased the dose rate of beams irradiating other sites with the aim to reduce the overall treatment time (45).This solution could be easily implemented as a further improvement of our approach. At the last follow-up, we did not observe any lung toxicity likely due the fulfilling of the mean dose constraint on the lung. However, to further reduce the possible long-term effects, we plan to incorporate the reduction of dose rate of beams irradiating the thorax.

## Conclusions

Implementing a dedicated couch for myeloablative TBI is feasible in any LINAC bunker. Moreover, TPS-based dose calculation enables more accurate and homogeneous dose distribution, considering the impact of the size and shape of lung blocks placed on the block tray or directly on the patient skin. Our approach based on a couch with an on-board EPID coupled with a modern LINAC, enables the accurate positioning and image verification of 3D-printed patient-specific lung blocks, while reducing the overall TBI set-up and delivery time. Finally, using 3D-printed patient-specific lung blocks have the potential to personalize the treatment and to eventually refine the shape of the blocks, making them extremely versatile.

## Data availability statement

The raw data supporting the conclusions of this article are available from the corresponding author upon reasonable request.

## Ethics statement

This study was reviewed and approved by the Ethical committee CE-AVEC 1065/2020/Oss/AOUBo. Written informed consent was obtained from all participants for their participation in this study.

## Author contributions

SS: Conceptualization, methodology, data curation and analysis, and writing–original draft. GP: Conceptualization, methodology, data curation and analysis, and writing–original draft. MS: data curation and writing–original draft. LC: methodology, writing: draft preparation, review, and editing. BB: data curation, writing–review and editing and resources, conceptualization, writing: review and editing. IA: data curation, writing–review and editing and resources. SC: data curation, writing–review and editing and resources. GC: data curation, writing–review and editing and resources. PW: data curation, writing–review and editing and resources. AM: supervision, resources, writing: review and editing. EM: supervision, resources, writing: review and editing. LS: Conceptualization, methodology, data curation and analysis, supervision, resources, writing: review and editing. All authors contributed to the article and approved the submitted version.
