# Supplementary material for: Improving total body irradiation with a dedicated couch and 3D-printed patient-specific lung blocks: A feasibility study
Source: Front Oncol. 2023 Jan 19;12:1046168. doi: 10.3389/fonc.2022.1046168 (PMC9893493; doi:10.3389/fonc.2022.1046168)
Supplement: Supplementary file 1 [file DataSheet_1.pdf]

Treatment Planning System implementation (Linac and dedicated planning CT)

The beam model commissioning of a TBI machine in the Pinnacle3 Treatment Planning System (TPS) v.16.2 (Philips Medical Systems®, 61 Fitchburg, WI) requires the acquisition of beam data in the form of profiles, depth doses, and output factors(OFs). In our center the measurements were performed on a VERSA HD Linac (Elekta, Stockholm, Sweden) using a Water Tank PTW MP3 (PTW, Freiberg, Germany). The water tank was placed on a trolley (with wheels) and centered using the two LINAC lasers installed in the bunker lateral walls and an additional ad hoc laser (see **Figure 1**) installed in the bunker ceiling, because the LINAC laser installed in the wall in front of LINAC gantry is blocked by the interposed Elekta couch . No beam spoiler was used for commissioning measurements. The water level was set to an SSD specific for our couch (SSD_c) of 170cm, as the expected maximum thickness of our patients in order to implement the TPS for an accurate calculation of absorbed dose distribution to the patient placed on the TBI couch. The OFs were determined to allow absolute dose calculations in Pinnacle. OFs measurements were performed using the above water tank at: 3x3, 5x5, 10x10, 15x15, 18x18, 20x20, 30x30, and 40x40cm^2 field size (defined at SSD =100cm i.e., SSD_{100}) using a standard 0.6cc Farmer chamber (PTW 30013, Germany) placed at SSD_c.

The machine OFs at extended SSD (i.e., at SSD_c) were calculated following the TG-51 protocol [19] as follows:

$$OF(\mathrm{SSD}_c, YxY, d = 10cm) = \frac{Measured\ dose\ (SSD_c, YxY, d)}{Measured\ dose\ (SSD_{100}, 10x10, d = d_{max})} * \left(\frac{1cGy}{MU.}\right)$$

where SSD_c indicates the SSD and YxY the field size at SSD=100cm projected at the extended field, while 10x10cm^2 is the reference field at SSD=100cm. The absolute dose per MU measured at SSD_c was divided by the machine output at standard SSD_{100} to account for variations in nominal machine output (1cGy/MU). Percentage depth doses (PDDs) and beam profiles were acquired for various field sizes with PTW Semiflex 31002 and PTW Semiflex 31010 as reference. PDDs were collected for beam size of 3x3, 5x5, 10x10, 15x15, 18x18, 20x20, 30x30, 40x40cm^2 at SSD=100cm. Profile scans were collected for the above field sizes at depths of maximum dose (i.e., 1.4 cm) and at 1.5, 5, 10, and 20 cm. An agreement between measured and calculated PDD and profiles ≤2% was considered appropriate for the TBI beam modeling. End-to-end testing was performed for the treatment simulation, planning, and delivery processes using an *ad-hoc* homemade phantom of water-equivalent plastic, polystyrene, and plexiglass slabs. The phantom was designed to reproduce the body densities as reported in **Figure 6**. Gafchromic EBT3+ film (Ashland, Wayne, NJ, USA) was placed axially between phantom slices in the thorax under the printed lung blocks. Two Farmer chambers were used to measure the planned dose at the phantom hemi-thickness at the head, thorax, abdomen, thigh, and leg level and at 1.3 cm under the entrance and exit of the phantom surface. The distance of 1.3 cm indicates the position of the center of the Farmer chamber insert in the chamber adaptation plate included in the slab phantom. A difference between measured and calculated doses within 2% was considered adequate to verify the beam model and the patient-specific quality assurance (QA). The measurements were performed according to the AAPM report #17 recommendations on phantom dosimetry[20]. No backscattering correction was applied in the latero-lateral direction since all the patients were smaller than the phantom in that direction.

Patient's setup imaging conversion

The image acquisition is performed using the dedicated imaging console PC, exported in .his* format, converted in .jpeg* using an in-house developed MATLAB GUI, and imported into the iViewGT imaging software (Elekta, Stockholm, Sweden). The image in .his* format was exported in a shared folder to be imported into the GUI. The user must import a DRR DICOM file (from which patient

information are extracted), select the treatment fraction, and, for each fraction, the number of current image acquisition. The .his* file is then imported, and a .csv log file is automatically created (or updated) with acquisition and patient information. the .his* file is converted into a .jpeg* image, visualized and saved in a shared folder.

	Mean±SD (Gy)	Min (Gy)	Max (Gy)
**Left Lung**	1.65±0.15	1.32	1.82
**Right Lung**	1.66±0.12	1.39	1.79
**Body**	2.01±0.02	1.96	2.04
**Body-Lungs**	2.02±0.03	1.97	2.07
**Heart**	2.04±0.05	1.93	2.11
**Liver**	2.01±0.03	1.96	2.08
**Right clavicle**	1.92±0.14	1.73	2.26
**Left clavicle**	1.91±0.14	1.62	2.24

Table S1: Mean doses (Gy) obtained from TPS measurements to the whole body minus Lungs, Lung, and collarbones. SD=standard deviation

PT #	S1	P1	S2	P2	S3	P3	S4	P4	S5	P5	S6	P6
**1**	4	2	1	5	1	1	1	1	2	1	1	1
**2**	3	3	2	2	2	3	1	3	1	2	1	1
**3**	2	3	3	2	2	3	2	1	1	2	3	2
**4**	1	2	3	2	1	2	2	2	2	2	2	2
**5**	3	1	2	2	3	2	3	2	2	1	2	2
**6**	3	3	4	3	1	4	2	1	1	3	1	2
**7**	4	4	3	1	2	2	2	1	3	3	1	2
**8**	2	3	4	3	2	2	2	4	4	2	2	2
**9**	2	4	2	2	1	3	2	2	3	2	3	2
**10**	2	1	2	1	1	1	2	1	2	2	2	2
**11**	2	1	3	2	1	3	1	3	2	3	2	1
**12**	1	2	1	1	1	1	2	1	2	1	2	2
**13**	2	2	5	2	2	1	3	1	2	2	3	2
**14**	1	1	1	1	1	1	1	1	2	1	1	1
**15**	2	2	1	3	1	4	3	1	3	2	1	1

Table S2: Number of setup images collected before each treatment per patient.

Sx (x=1,2,3,4,5,6): supine position in the x-th fraction

Px: prone position in the x-th fraction

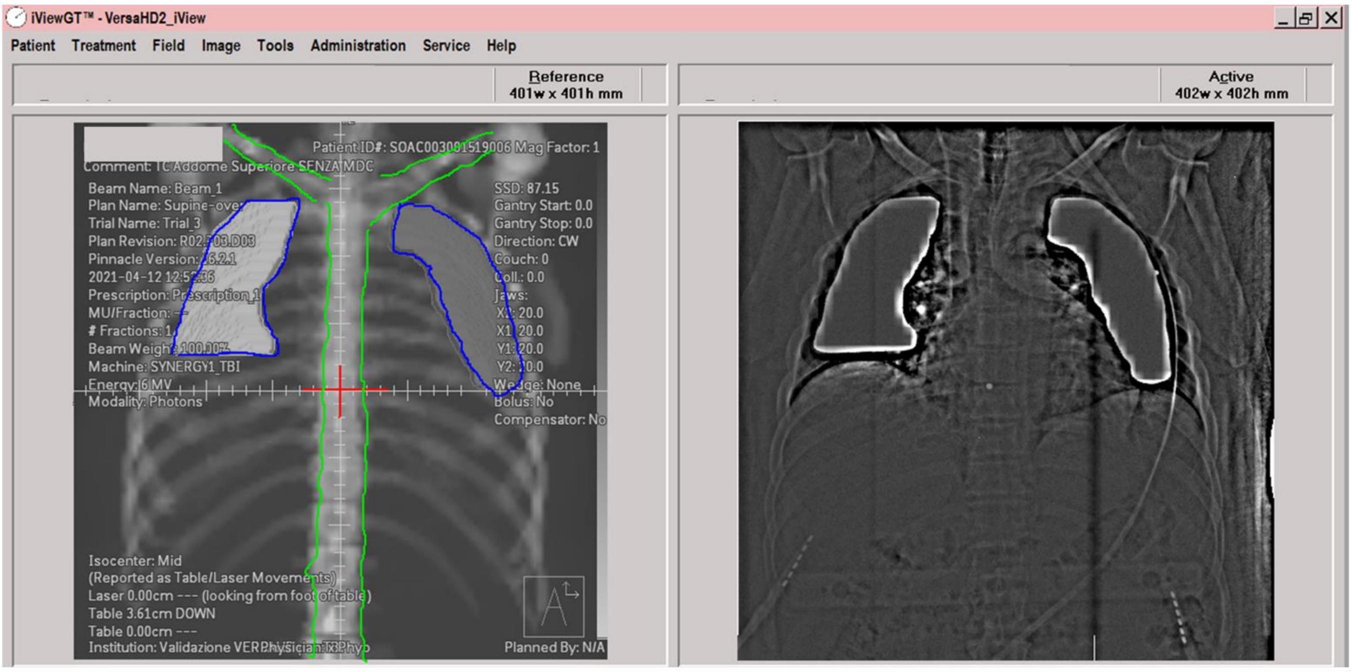

Figure S1. Example of comparison between the DRR and the set-up image acquired with the TBI couch-dedicated EPID panel.
